# “I need personal experiences or some sort of documentation”: a qualitative study on where people with multiple sclerosis seek information on dietary and herbal supplements

**DOI:** 10.1186/s12906-021-03377-0

**Published:** 2021-08-21

**Authors:** Sofie Bergien, Clara M. Petersen, Marie Lynning, Maria Kristiansen, Lasse Skovgaard

**Affiliations:** 1 The Danish Multiple Sclerosis Society, Poul Bundgaards Vej 1, Valby, Denmark; 2 Department of Public Health and Center for Healthy Aging, University of Copenhagen, Øster Farimagsgade 5, 1353 Copenhagen K, Denmark

**Keywords:** Dietary supplements, Herbal supplements, Multiple sclerosis, Information seeking

## Abstract

**Background:**

The use of dietary and herbal supplements (DIHES) is widespread among people with multiple sclerosis (PwMS). PwMS are a highly informed patient group, and they use several types of sources to seek information on subjects related to their disease. However, it is still unknown where PwMS seek information about DIHES. It is important that PwMS make decisions about DIHES based on accurate, useful and accessible information. Therefore, the aim of this study was to explore where PwMS seek information on DIHES and how they experience and engage with this information.

**Methods:**

Semi-structured interviews were conducted with eighteen PwMS using DIHES. Participants were selected from a cross-sectional survey. Diversity sampling was used, based on relevant characteristics such as gender and number of DIHES used during the past 12 months. The interviews were conducted face-to-face or over the telephone and lasted between 30 min and 1 hour. The interviews were recorded, transcribed verbatim, and analyzed using thematic network analysis in NVivo 12 Pro software.

**Results:**

Three main themes emerged in the analysis: i) engaging with healthcare professionals (HCPs) regarding DIHES, ii) social networks as a source of information regarding DIHES, and iii) reliance on bodily sensations. Most participants navigated all three types of sources. All participants had at some point discussed DIHES with an HCP. Information from HCPs was considered reliable and valuable, but HCPs were viewed as uncommitted to the dialogue about DIHES. Recommendations from others were often the driver of decisions regarding use of DIHES. However, the information from PwMS’ networks could be overwhelming and difficult to navigate. Finally, PwMS relied on their own experiences regarding DIHES and let their bodily sensations guide their use of DIHES.

**Conclusions:**

Participants often rely on all three types of information sources to create a nuanced and comprehensive information base. However, PwMS may feel overwhelmed or confused with all the information they have gathered. These findings indicate the need for better guidance for PwMS concerning DIHES and an openness among HCPs to engage in dialogue.

**Supplementary Information:**

The online version contains supplementary material available at 10.1186/s12906-021-03377-0.

## Background

Multiple sclerosis (MS) is an autoimmune, neurological disease affecting 2.3 million people worldwide [1]. People with MS (PwMS) may suffer from a variety of symptoms, including motor impairment, pain, fatigue, and cognitive challenges [2, 3]. Thus far, MS is incurable, and medical treatment only aims to modify the progression of the disease or to manage MS-related symptoms [4, 5]. It has been argued that due to dissatisfaction with conventional treatment, and a desire to feel more involved in one’s own treatment, many PwMS use products such as dietary and herbal supplements (DIHES) [6–8]. DIHES can be defined as products that are taken orally and contain vitamins, minerals, enzymes, herbs, animal ingredients, or amino acids [9]. In Denmark, as well as in other countries, DIHES are sold over the counter and are therefore easy for consumers to access and potentially self-administer [10, 11]. A recent Danish study found that over 80% of PwMS in Denmark had used DIHES within the previous year; a tendency also seen in countries such as Germany, the United States, and the United Kingdom [8, 12–14]. PwMS often used DIHES concomitant with conventional treatments, which has led to concerns about potential drug interactions [6, 8, 13, 15]. Several types of DIHES that are used frequently by PwMS, such as echinacea and cannabis, have the potential to cause undesirable interactions if they are used concomitant with medicine often prescribed to PwMS by healthcare professionals (HCPs) [13, 16]. However, only a small number of PwMS who use DIHES are aware of this potential risk [8]. The risk of drug interactions has led to a call for awareness and the need for more information on proper and safe use of DIHES among PwMS [8, 13, 15, 16].

PwMS are generally perceived as a highly informed patient group who use several types of sources to seek information [17–20]. Depending on the subject, PwMS navigate sources such as family and friends, the internet, HCPs, national MS societies, books, and magazines [21]. Despite the high prevalence of DIHES use among PwMS, the nature of their information-seeking behavior regarding DIHES is limited. Only two existing studies investigate information-seeking behavior among PwMS. A Danish study from 2014 found that PwMS use their bodily sensations as a risk assessment tool, but they rarely discuss their use of DIHES with their HCPs. However, this study did not investigate which other information sources PwMS use to obtain information on DIHES. An Italian study also found that PwMS rarely disclose their use of DIHES to HCPs, but that advice more often is sought from an herbalist or acquaintance [6]. However, this study was based on data from a survey and does not provide a deeper understanding of why PwMS choose to obtain their information on DIHES from one source instead of another. To ensure that PwMS can access the proper information on DIHES in the future, it is necessary to have a better understanding of their information-seeking behavior in this area. In this study, we therefore aim to investigate where PwMS seek information on DIHES and how they experience and engage with this information.

## Methods

### Study design

The present paper is based on a larger sequential explanatory mixed methods study, carried out from February 2019 to June 2020. The study consisted of a cross-sectional survey [8], a systematic literature review [16] and qualitative in-depth interviews. The results shared in this paper are based on data from the interviews, the primary aim of which was to explore information-seeking behavior among PwMS related to their use of DIHES.

### Selection of participants

**Fig. 1:**
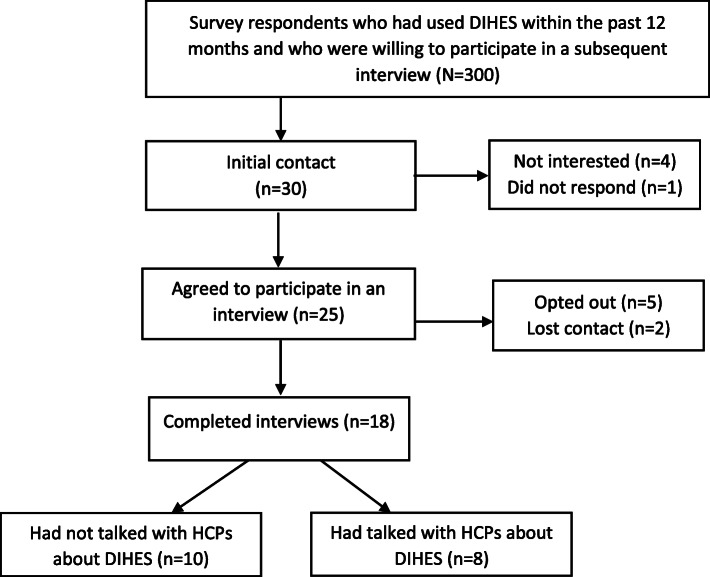
Study flowchart

Participants were recruited from the participant sample of the cross-sectional survey carried out among members of the Danish MS Society [8]. As part of the survey, all respondents were asked if they were willing to participate in a subsequent individual interview about their use of DIHES. In total, 354 out of 380 respondents (93.2%) agreed to be contacted again, and participants were purposely recruited from the sample of respondents who reported using DIHES within the past 12 months (*N* = 300). Diversity sampling was used according to gender, type of MS, level of education, as well as number and types of DIHES used during the past 12 months. Participants were strategically selected to represent a maximum variation on these variables. Further, the participants were recruited such that about half had talked with their HCP, such as medical doctors, nurses, pharmacists, and dietitians, about using DIHES, and half had not. In total, 30 PwMS were invited to participate in the interviews. The invitations were sent by e-mail and included information on the purpose of the interview and a date for a phone call to schedule the specific time and place for the interview. Eighteen of the selected participants ultimately participated in an interview (Fig. 1). Table 1 summarizes the participant characteristics. Pseudonyms are used for all the participants in this paper.

**Table 1:** Characteristics of the participants (*n* = 18)

Participant^*^	Age	Sex	YSMD^a^	Type of MS^b^	Symptoms^c^	Level of education^d^	MS clinic^e^	Dietary and herbal supplements^f^
Charles	60–69	M	26	RRMS	8	3	1–2	Cranberry and Psyllium fiber
Sophie	30–39	F	3	RRMS	12	2	3–6	Vitamin D, Vitamin B12
Charlotte	50–59	F	16	SPMS	6	2	< 1	Magnesium, Unknown supplement
Emily	30–39	F	37	PPMS	7	1	1–2	Vitamin D, Multivitamins, Fish oil, Iron
Margaret	50–59	F	10	RRMS	9	3	1–2	Multivitamins, Vitamin B, Calcium with Vitamin D, Fish oil, Alpha-lipoic acid, Evening primrose oil
Elizabeth	50–59	F	16	RRMS	10	3	1–2	Cranberry, Vitamin D, Probiotics, Fish oil, Calcium
Sarah	40–49	F	13	PPMS	10	3	< 1	Vitamin D, Vitamin B, Multivitamins, Omega-3 (EPA and DHA), Magnesium
James	60–69	M	19	PPMS	2	2	1–2	Multivitamins, Calcium with Vitamin D, one supplements containing fennel seeds, chamomile flower and peppermint leaves
George	40–49	M	23	Don’t know	3	2	> 6	Vitamin C, non-prescription cannabis
Emma	30–39	F	1	RRMS	10	2	1–2	Multivitamins, Fish oil, Magnesium, Psyllium fiber, lactase enzymes
Victoria	40–49	F	10	RRMS	7	3	1–2	Vitamin D, Vitamin C, Vitamin B12, Vitamin B3, Multivitamins, Omega-supplement, Coenzyme Q10, Calcium, Magnesium, Zinc, Selenium, Turmeric
Oscar	20–29	M	1	RRMS	0	3	3–6	Vitamin D, Fish oil
Harry	70–79	M	1	SPMS	8	3	< 1	Calcium, Magnesium with Vitamin D, Fish oil
Lauren	40–49	F	8	RRMS	6	1	1–2	Vitamin D
William	40–49	M	3	RRMS	10	2	3–6	Multivitamins, Calcium, Fish oil, Unknow supplement, two types of non-prescription cannabis
Joe	50–59	M	15	PPMS	10	1	No	Calcium, non-proscription cannabis oil
Thomas	50–59	M	33	SPMS	7	2	3–6	Vitamin D, Vitamin C, Multivitamins, Fish oil, Zinc, Boron, Turmeric, non-proskription cannabis oil
Michael	40–49	M	6	RRMS	6	3	> 6	Vitamin D, Calcium

^*^ Pseudonyms are used for all the participants in this paper

^a^*YSMD* Years since MS diagnosis

^b^ Type of MS: RRMS (relapse-remitting MS), SPMS (secondary progressive MS) and PPMS (primary progressive MS)

^c^ Self-reported number of MS-related symptoms – Including: pain, muscle spasm, fatigue, walking impairment, sexual dysfunction, abnormal sensation, muscle weakness in arms and legs, vision problems, bladder and bowel problems, cognitive challenges, emotional dysfunction, depression and anxiety, sleep problems, dizziness

^d^ 1) Below high school, 2) High school or vocational education, 3) Bachelor’s degree or more

^e^ Number of yearly appointments at a MS clinic with a neurologist or an MS nurse

^f^ Dietary and herbal supplements used within the past 12 months

### Data collection

Interviews were conducted by the authors SOB and CMP between September and November 2019. All interviews were carried out face to face or over the phone. The face-to-face interviews were conducted in locations convenient for the participants. As a result, 11 participants were interviewed in their home and 3 in a meeting room of the Danish MS Society. The remaining four participants were interviewed over the phone. In the methodological literature, it has often been argued that telephone interviews are not sustainable in qualitative research [22]. However, in some circumstances they can be an useful and relevant option, especially if they provide the opportunity to include people who otherwise would have been excluded from the study [22]. In this study, face-to-face interviews were replaced with telephone interviews in four cases, by choice of the individual participant, as they lacked the energy to carry out a face-to-face interview. Informed written and oral consent was collected from all participants before the interview began.

A semi-structured interview guide informed by the aim of the study, existing literature [23], and the survey results [8] were developed by author SOB and MK (See Additional file 1). Overall, the interview guide was designed to procure insight into four main themes: i) reasons for using DIHES, ii) accessing information on DIHES, iii) communication with HCPs about DIHES, and iv) knowledge about interaction between DIHES and conventional medicine. The interview guide consisted predominantly of open-ended questions, such as: “Would you like to start with saying a few words about what you think about using products such as vitamins, herbs and oils?”; “When did you last hear and/or talk to anyone about DIHES?”; and “Can you tell me about what you read/talked about?” Furthermore, each interview guide was informed by the participant’s individual answers in the survey. If participants in the survey answered that they had used calcium and vitamin D, they were asked, “Can you tell me about the time you started using vitamin D and calcium?”. Before the interviews, the participants were informed that DIHES were defined as products containing vitamins, minerals, enzymes, herbs, animal ingredients, or amino acids that are taken orally and can be bought without a prescription. This definition also covered DIHES that were not necessarily legal in Denmark but could be imported legally from other European countries or grown by the participants, such as cannabis. In order to assess the quality of the interview guide, it was reviewed after the first four interviews. Examining the transcriptions, the authors CPE and MK found that the guide provided a good framework for the interviews. After 18 interviews, the authors SOB and CMP found that a thematic saturation of data had been achieved, and the 12 individuals who did not accept the invitation were therefore not replaced.

The interviews lasted between 30 min and 1 hour. Data concerning background information (gender, age, level of education, type of MS, years since diagnosis, number of self-reported symptoms, number of yearly appointments at an MS clinic, and number of DIHES used in the past 12 months) was retrieved from the original survey.

### Data analysis

All interviews were recorded with the consent of each participant and transcribed verbatim by CMP. The interviews were analyzed using a thematic network analysis and taking an abductive approach [24]. The initial analysis was carried out by SOB. Before the coding of data, each interview was read in full to get a sense of the whole dataset. To guide the analyses, a coding framework was developed based on the subjects covered in the interview guide as well as on emergent themes identified in the preliminary reading of the interviews. Meaningful text segments were coded and organized using NVivo 12 Pro software. Subsequently, all text segments under each code were read, and subthemes emerged from the most salient and common codes. In total, 35 subthemes emerged, 9 of which were related to where PwMS search for information and how they experience and engage with this information (Table 2). Each subtheme was labeled to summarize the essence of the coded text segments attached to it. When all subthemes had been identified, CMP and MK read through the material to validate the text segments in each subtheme. The subthemes were then clustered into main themes in a thematic network by SOB. Based on the thematic network, subthemes as well as main themes were discussed in the research group, and the final network was refined and verified. No member validation was implemented, as it was assessed that it would be burdensome for the participants to participate in this process, given PwMS’ challenges regarding fatigue and cognitive function [25, 26]. The authors’ reflexivity was addressed continuously as part of the work and discussed in the larger study’s project group. In addition to the authors, the group consisted of one neurologist, one person with MS who used DIHES, one biopath and naturopath, and two researchers working with research in natural products. The composition of the group made it a useful environment for discussing the use of DIHES as well as the members’ attitudes and biases toward such products. The use of (…) in quotes symbolizes removed text that is irrelevant for the understanding, and words surrounded by [...] have been added by the authors to improve the understanding of the quote.

**Table 2:** Overview of main themes and subthemes

Main themes	Subthemes
Engaging with HCPs regarding DIHES	• HCP being uninvested when the dialogue about DIHES is initiated by the patient • PwMS are taking a passive patient role when the dialogue about DIHES is initiated by the HCP • Information from HCPs is objective and standardized
Social networks as a source of information regarding DIHES	• Reliance on the experiences of others • Information from social networks is concrete and relatable • Unsolicited recommendations from network are difficult to navigate
Reliance on bodily sensations	• Every person is unique • Recommendations are evaluated through bodily sensations • Difficult to navigate body sensations

### Ethical approval

In Denmark, only studies including biological material from humans can be approved by the National Committee on Health Research Ethics [27]. For this study, therefore, no approval was needed. However, the study adhered to the EU General Data Protection Regulation and ethical principles for medical research as presented in the Declaration of Helsinki.

## Results

### Participant responses and characteristics

Participant characteristics are presented in Table 1. The participants were primarily between 40 and 60 years old, with the youngest participant being 28 and the oldest 72. Half of the participants were women, and half were men. Most of the participants had been diagnosed with MS more than 3 years earlier and were, at the time of the interview, diagnosed with relapse-remitting MS. The participants had a high self-reported burden of MS-related symptoms, as most of them experienced five or more symptoms. Three of the participants were educated below high school level, seven had a high school or vocational education, and eight had a bachelor’s degree or higher as their highest level of education. The participants lived in both urban and rural areas of Denmark. All except one had been in contact with a Danish MS clinic within the past year. Most had been using between two and four different kind of DIHES within the last 12 months. The survey data showed that 10 of the participants had not talked with their HCPs about the DIHES they had used. However, the interviews revealed that all participants had at some point discussed DIHES with a HCP.

### Sources used to get information regarding DIHES

Table 2 summarizes the three main themes and their subthemes: 1) Engaging with HCPs regarding DIHES; 2) Social networks as a source of information regarding DIHES; and 3) Reliance on bodily sensations. Almost all the participants explained that they navigate between sources to collect the information they need about DIHES. In the following section, the three main themes will be presented.

### Theme 1: engaging with HCPs regarding DIHES

#### HCP being uninvested when the dialogue about DIHES is initiated by the patient

Most participants explained that they had at some point initiated a conversation about DIHES with their HCP by mentioning what kind of DIHES they were using or asking their HCP what to use. Many participants explained that they found their HCP’s reaction to be passive. While their HCP may have acknowledged their use of DIHES, they refrained from entering a dialogue or providing the feedback the participants were hoping for. One participant said that he “…*has the feeling that it goes in one ear and out the other.*” Another participant explained that because of her physiotherapist’s lack of willingness to engage in discussions on DIHES, she would no longer ask her for advice and would instead search for information on DIHES elsewhere:

*“I am really happy with my physiotherapist – I have known her for many years. (…) She nods in recognition and tells me that it was good I figured it out. But for this reason, I no longer tell her about what I do, because I don’t need recognition. I need competent feedback, and I don’t feel like I get it there. (…) If I want competent feedback, then I need to discuss it with someone who knows something about it. And that is the people who experiment with and use the natural methods.” – Margaret*

#### PwMS are taking a passive patient role when the dialogue about DIHES is initiated by the HCP

Despite experiences with a lack of dialogue resulting in participants not raising questions and concerns about DIHES in encounters with HCPs, several participants nevertheless mentioned HCPs as a source for information about DIHES. Many of the participants seemed to be taking a passive role and letting their HCP decide when and how to talk about the subject:

*” Yes, it is something the doctors have seen in the [blood] samples and which they think could be beneficial, such as extra iron, extra D-vitamin and so on. (…). It is the doctors who bring it [*DIHES*] up. I wouldn’t at all want to put words in their mouths. As I said, they look at the samples and then they tell me, well you are lacking some of this, maybe you should have some of that.” – Emma*

The participant quoted above explained that her doctor recommended she use vitamin D, a product frequently cited by participants as having been recommended by their HCP. Besides vitamin D, multi-vitamins, vitamin B, cranberry, magnesium, iron, and psyllium were mentioned by the participants as products recommended by their HCP.

Some of the participants expressed that they were not always sure why they should use the products their HCP had recommended. For example, a woman explained that her neurologist did not explain why she needed to use vitamin D:

*“It was the neurologist who said that since I have MS, they recommend that I take it [Vitamin D]. If it is because they are uncertain about whether multiple sclerosis derives from lack of some vitamin, I don’t know. But I take it because they tell me to. (…) He [the neurologist] told me [to use vitamin D] when I was newly diagnosed with MS. [At that time] you are in a state of crisis and you can’t handle more information of any kind. (…) I actually haven’t asked about what the various stuff [DIHES] do, other than I have been told to take it. So, I take it. And then I assume they have it under control”. – Sophie*

#### Information from HCPs is objective and standardized

Many of the participants found that the information provided by their HCP was based on objective measurements, such as blood samples. Thus, HCP recommendations regarding DIHES were in some cases associated with inconvenience:

*“The neurologists want to give me answers, but they need blood samples to know what I’m lacking and what I need. So, they ask for that. And then I need to go there for blood tests and so on. Then it becomes inconvenient and I don’t really want to do it. No, I don’t. Then I would rather use my network or the internet.” – Emma*

Other participants reported that the HCPs’ educational background directly inhibited their willingness to provide information on DIHES and that the HCPs lacked understanding of how it feels to live with MS. Both circumstances were experienced by some participants as a shortcoming in the way the HCP handled the subject:

*“I believe that doctors may be very qualified because they have studied for many years, but a lot of them don’t really know how we feel or how I feel. I experience that a lot. Because the things they tell me is what they know from theory. Evidence on this [DIHES] is very rare. But they always lump people together. I mean, they do not see me, right? They only see a person with MS sitting there. That’s my experience at least.” – Sarah*

Other participants, in contrast, explained that HCPs should not be expected to provide information regarding DIHES and that HCPs must adhere to what is supported by clinical studies and theories. A male informant explained that he expects the doctor to set the agenda during consultations and that information on DIHES is something one should acquire from other sources:

*I do not go to see the doctor to talk about something she doesn’t want to talk about or have any knowledge about or doesn’t want to listen to. I visit the doctor because she is the specialist. So, what we talk about is what she thinks we should talk about, and she doesn’t want to talk about some alternative treatment. I think they want to discuss the things they are educated to be talking about. The things they tell me must be substantiated by their education and their knowledge. And I actually expect that when she tells me to take something, then it’s because she knows that it will be good for me. It would be wrong if she advised me to do something that she herself couldn’t vouch for. I would not expect her to do that, and that is not the reason I go there. If I wanted that, then I could easily find the information I needed in a group and by talking to others, but I wouldn’t visit the doctor or hospital. – Charles*

### Theme 2: social networks as a source of information regarding DIHES

#### Reliance on the experiences of others

All but two participants mentioned that they had used their social networks as a source of information about DIHES. In the interviews, social networks appeared as both online networks and real-life networks. Many participants explained that they engaged with online forums such as Facebook or private blogs to obtain information from other patients, but family, friends, and other patients they had met in the healthcare system were also mentioned as sources of information about DIHES. One participant explained that recommendations regarding which DIHES to use and how to use them did not necessarily have to come from other PwMS but could also come from patients with other diseases characterized by similar symptoms. Some of the participants mentioned that people with cancer could be particularly beneficial to consult regarding use of cannabis. One participant phrased it this way:

*One of my mom’s friends has battled cancer and used cannabis in this context. She talked to me about her experience with using cannabis oil. She used, you know, pure top shoots, it was tough stuff, right? And she said that with her disease, it is of course something completely different, but that she wouldn’t have survived without it. And she also recommended I try it.” – William*

Another participant spoke about how this kind of information sharing between people has become easier over time:

*The peer-to-peer networks have been improved significantly and become more easily accessible. A huge body of knowledge exists in patient communities and from people living with diseases. (…) For instance, regarding the topic of how to control your blood sugar in relation to alcohol consumption. The standard answer from the doctors would be “you should hold back and be aware that your blood sugar will drop.” But if you want specific knowledge about certain kinds of beer and how to do it, then other people have created exact tables and suggestions on how to do it that they can share. Then it gets very specific to diabetes and it is stuff like that that needs to be shared in those forums. – Michael*

#### Information from social networks is concrete and relatable

Many of the participants talked positively about the information they could get about DIHES from peers in real life or online. When asked why that kind of information was especially valuable, they explained that compared to information given by HCPs or found in non-anecdotal articles, it was much more concrete and reliable:

*Yes, definitely. I think it is often more rewarding to talk to other MS patients about what to do than to seek advice from the healthcare system – not only about [natural] medication, but also regarding exercise machines or specific exercises and other stuff. Because personal experiences are important to me. I rely on what people tell me; for instance, if someone I knew and trusted told me about a good craftsman, then I would hire him based on their words. And the same goes for natural medicines, but not just out of the blue. An article in the newspaper saying, “try ginger” – that wouldn’t be enough. I need personal experiences or some sort of documentation. – William*

One participant explained that he considered others’ accounts of their experiences with DIHES to be valid documentation regarding effect:

*Well to me, I am also a pragmatist unfortunately. If there is a documented effect – and to me a documented effect can very well be based on conversations I have had with MS patients over the phone who would share their experiences. Or when I’ve been sitting in waiting rooms and someone I met would tell me it worked for them or that it took the edge off or something. Then it would interest me! – William*

#### Unsolicited recommendations from networks are difficult to navigate

In contrast, some participants emphasized that information from peers could be overwhelming and difficult to navigate. Especially when newly diagnosed or experiencing new symptoms, the participants explained that social networks offered unsolicited recommendations regarding different kinds of DIHES that might help them. A woman explained that when she was diagnosed with MS, her friends and family recommended she use DIHES that would boost her immune system. It took some time before she was able to refuse and tell them it actually was not good for her:

*I have an extremely good network, so, everybody was like…. I mean you get sad when you receive that kind of news [an MS diagnosis]. There was a lot to overcome [for me]. But after that, when I figured out what it all was about, I had to tell people that it was really nice of them [to try to help] but that the issue is that my immune system is a little over-active or a little different. Instead of explaining everything every time, since I didn’t always have the energy to do so, I just told them my immune system was good. Then that was cleared, and I didn’t have to get any more advice of any kind like use krauterbleut, or biostrat and all sorts of other natural things. Some things I have chosen to use, and other things not to use. – Margaret*

### Theme 3: reliance on bodily sensations

#### Every person is unique

Many of the participants expressed that even though PwMS might experience the same kinds of symptoms, they believed that the effect of specific DIHES would differ from person to person. The participants felt that it was ultimately up to the individual to assess how a specific product would work for them. One participant explained that her body – and thus her MS – differed from others’, and that people should evaluate the effect of DIHES on their own body individually:

*Because the body is different from person to person. I am a very strong person, and so it is difficult to compare my multiple sclerosis to others’. So, I definitely think other people should take that natural medicine and try to take it only two months or three, maybe six months or a year, and then they can evaluate whether it will help them. – Emily*

#### Recommendations are evaluated through bodily sensations

Most of the participants expressed that they used their bodily sensations to evaluate the effects and side effects of specific products they had been recommended. As one participant explained, the lack of experienced effect on her own body led her to stop using the DIHES she had read about in a book:

*I have tried a lot of things, and I don’t feel a difference (…) I have the book, I can’t remember what the book is called… It said a lot about what was good for one’s cells and brain. So I tried folic acid, right? And I’ve been taking it for a while and I can’t feel anything, so I’ve simply dropped it. – Charlotte*

Furthermore, many of the participants explained that they often supplemented and validated information about DIHES from HCPs or peers based on their own bodily sensation. A male participant explained that his nurse recommended magnesium and psyllium fiber, but he only continued using fiber because magnesium did not make a difference for him in his everyday life:

*Yes, psyllium fiber [I still use]. Magnesium not so much. I didn’t take that a lot, because I don’t think it really made a difference in my everyday life. I didn’t feel that it changed my pain or nerve disorders much, so no, I don’t think it actually worked. Psyllium fiber worked. But then again, it worked on the current problem [digestive problems], whereas nerve pain is a more general problem. The doctors themselves said that I should not count on it going away, so I don’t think that it [magnesium] is going to help no matter how long I take it. – Emma*

#### Difficulty in navigating bodily sensations

Some of the participants expressed that it could be difficult to rely on bodily sensations to inform their decisions about DIHES. They indicated that especially in the early stages of their disease, it took some time for them to get to know their body and the patterns of their disease. One participant explained that the period after a relapse, when symptoms ease, he could be tricked into believing that it was the DIHES that were helping him feel better:

*No, but in the beginning, I also read a lot, for example in the [Danish MS Society’s] magazine, and there were often success stories. Back then I couldn’t see that after you’ve had a relapse, you have some peaks where you’re up [feeling better], and that’s what it said in the magazine. (…) If you don’t have the experience [of feeling better after a relapse], then you will be fooled [because you think it is the new products you are using, a new training exercise or something else that leads to the improvement]. I did that in the beginning as well because I didn’t know that much about it. – Thomas*

Furthermore, some of the participants explained that concomitant use of different DIHES could make it difficult for them to navigate their own bodily sensations:

*And that’s the challenge (…) When you take these supplements, you usually take several different products; it’s a bit like with medicine. So, what the hell, what works with what. And that is why I sometimes stop, because in reality I can’t figure out where to invest my money. – Sarah*

## Discussion

### Main findings

This study investigated how PwMS seek information about DIHES and how they experience and engage with this information. The results of the study indicate that information is mainly sought from three different types of sources: HCPs, social networks, and bodily sensations. According to the participants, all three sources of information entail certain advantages and disadvantages. Information from HCPs was considered reliable and valuable, but at the same time, HCPs were viewed as uncommitted to the dialogue about DIHES if it went beyond what they considered to be evidence based. Recommendations from social networks were considered valid and important and were emphasized as a main driver of decisions regarding use of DIHES. However, the amount of information from social networks was also articulated as being overwhelming and difficult to navigate. Finally, participants emphasized the importance of relying on their own experiences regarding DIHES – in other words, leveraging their own bodily sensations in their choices. However, participants also expressed that relying on their bodies carried some uncertainty, as changes to bodily sensations may have been explained by other factors, such as disease fluctuations. The results of the study indicate that participants often combine all three sources of information rather than relying on only one. Such combination serves the purpose of achieving a nuanced and comprehensive information base. The study did not find that complementary and alternative practitioners were considered an essential source of information among the participants.

Only a few previous studies have investigated information-seeking behavior regarding DIHES among PwMS [6, 28]. Most studies have reported on the use of DIHES as a part of complementary and alternative medicine (CAM) in general [12, 29, 30]. CAM may be defined as a non-mainstream practice used together with or instead of conventional medicine [31]. Based on this definition, DIHES can be used as CAM, but can also be part of the conventional healthcare system. The present study also found that DIHES are at times recommended by the HCP and are at other times used together with or instead of conventional treatments. Whether DIHES should be perceived as CAM or conventional treatment is often a question of interpretation and may depend on the diagnoses and/or treatment regimes in question. However, due to the lack of comparative literature, the findings in this study will be discussed in comparison with literature covering information-seeking behavior regarding CAM or literature covering other patient groups’ information-seeking behavior specifically regarding DIHES.

### Information overload

The results of the present study indicate that PwMS use a wide variety of external sources to gain information about DIHES. These sources include recommendations from HCPs as well as recommendations from other patients either directly or via channels such as online forums, blogs, and magazines. These findings support earlier studies that have described PwMS as a highly informed patient group who use a variety of sources to gather information on various subjects, including DIHES [6, 17–19]. The present study’s results further suggest that PwMS do not necessarily find it difficult to navigate between the different sources. Rather, they use these different kinds of information to establish a more complete picture of which DIHES to use when and how. For instance, the participants might receive a recommendation to use DIHES from their HCP, or from someone in their social network, but they rely on their own body’s signals to evaluate whether the specific product works for them.

However, the findings also suggest that the large amount of information may be overwhelming for some patients. Some participants indicated that, especially when newly diagnosed, unsolicited recommendations from social networks can be challenging to navigate. This finding is in line with a previous study by Synder et al., who argued that PwMS can experience information overload when gathering information on MS treatments from the internet [32]. The present study contributes to the research field by showing that information overload can also occur when patients rely on other sources, such as social networks outside the internet. It has previously been found that access to too much information may have a negative impact on patients’ psychological well-being [33]. Supporting patients by providing individually tailored information might help them act on this information [34]. However, when information comes from sources such as social networks, it is not possible to control the information, and it has been argued that more research is needed on initiatives that could help people manage health information overload [35]. This perspective is especially relevant for PwMS due to the cognitive challenges that often come with the disease and which may inhibit the patient’s ability to process and assess large amounts of information [3].

### What constitutes useful knowledge?

In the existing literature, it is reported that people, including PwMS who use DIHES, often receive their information on DIHES from sources such as family, friends, books, magazines, or the internet [6, 36–38]. The present study finds that from the participants’ perspective, knowledge regarding DIHES did not have to be scientifically based to be perceived as valid and useful. These findings are supported by earlier studies among PwMS and the general population. A Danish study of PwMS’ views on the risks of negative interactions between herbal medicines and conventional drug therapies found that types of information other than traditional evidence-based information were seen as valuable by PwMS [28]. In their study from 2018 among the general population in Germany, Welz et al. found that information sources such as family members were often considered more important than recommendations from HCP [39]. The present study finds that the participants’ own experiences, as well as others’ experiences, were considered just as important as scientific evidence, and that some people perceived experience based and anecdotal knowledge about DIHES to hold just as much epistemological value as evidence-based knowledge. In their review of studies exploring cancer patients’ decision making regarding CAM in general, Weeks et al. found that what was accepted as evidence varied greatly from person to person and that this distinction depended on individual factors, such as underlying beliefs and values, experience with CAM, and the stage of the disease [40]. Smith et al. similarly reported that, while having evidence of the efficacy of CAM interventions for autism spectrum disorder was viewed as being essential for guiding decisions, their participants had different views on what constituted evidence, and they combined different types of evidence to guide their decisions regarding CAM use [41]. The present study finds that, regardless of whether DIHES is seen as being conventional treatment (recommended by HCPs) or CAM, participants combine different types of evidence to guide them in their decisions regarding DIHES.

### Difficulties in understanding bodily sensations

The participants in the present study indicated that they rely to a large extent on their own bodily experiences to guide them regarding use and validation of DIHES. A previous study among PwMS reported similar results, revealing that patients relied on their own bodily sensations to determine whether an herbal remedy had any negative effects [28]. Qualitative studies have found that people depend heavily on their own experiences with CAM to guide their decisions regarding choice of products. McClymont et al. found that patients with back pain relied on what the authors refer to as internal information – previously acquired knowledge or experience – to guide them in their choices about CAM therapies [42]. Hill-Sakurai et al. reported that women seeking CAM for symptoms related to menopause used “listening to one’s body” and noticing what “feels right” as guides. Similarly, parents of children with autism spectrum disorder describe a process of “trial and error” when using CAM with their child, using the child’s response as a guide to decide whether to continue or terminate the treatment [41]. The results of the present study indicate that this method is applied by users regardless of how the product in question has been recommended and regardless of whether the product in question is defined as CAM. While relying on one’s own experiences may be a useful strategy, especially when evaluating the adverse effects of a treatment, the findings in the present study suggest that it may create additional difficulties for some patients. As some informants emphasized, the choice to rely on bodily signals and sensations may entail doubts and uncertainty. For example, it may be difficult to discern whether changes in symptoms or sensations are caused by DIHES or whether they are related to disease fluctuations or the effects of conventional medicine. This uncertainty may pose a burden for some patients, not least those in an early state of disease who do not yet fully understand how their disease is expressed physically. A recent study found that PwMS who were newly diagnosed were more likely to use DIHES [8], making it even more important to consider whether PwMS should be supported in how to interpret their bodily sensations to evaluate effects and side effects.

### Uninvested HCPs

All informants in the present study reported having brought up the use of DIHES with at least one HCP or had tried to get a product recommended by an HCP. Many participants found HCPs to be mostly passive and uninterested in engaging in a dialogue about DIHES when the dialog was initiated by the patient. While some informants did not expect HCPs to engage in dialogue about DIHES, others indicated that they would like HCPs to show more interest in and provide advice about DIHES. Studies regarding information-seeking about CAM have likewise found that informants value the advice of HCPs [41] and desire HCPs to be more informed about and open to dialogue regarding the use of CAM [43]. Similarly, Skovgaard et al. found that PwMS lose the desire to bring up the subject of herbal medicine with their medical doctor when the doctor shows no interest in the subject or has no knowledge about it [28]. A 2019 study on how HCPs in United Kingdom view herbal medicine, found that many HCPs do not feel they have enough knowledge or education to advise their patients on the subject [44]. However, dismissing a dialogue may have several negative consequences. If patients feel rejected by their HCP when they bring up the subject of DIHES, then they may become less open in general, and important information may be lost. If patients do not share information about their use of DIHES with their HCPs, then negative interactions with conventional drugs or adverse effects of DIHES may not be detected, which may have consequences for patient safety [44]. These potential outcomes emphasize the importance of HCPs engaging in dialogue with their patients on subjects that may seem to be peripheral to conventional medical treatment.

### Strengths and limitations

Thirty individuals were invited to participate in the study, and 18 accepted. It cannot be ruled out that additional themes might have emerged from a larger sample. However, the 12 individuals who did not accept the invitation were not replaced as the authors found that a thematic saturation of data was achieved. PwMS often have a significant symptom burden and experience days with high levels of fatigue or pain. As the interviews took place at the most convenient location for each participant, and as the interviewers were flexible regarding time and date in order to accommodate the participants’ wishes and needs, the risk of excluding people due to lack of energy, or because they did not have an interest in DIHES and therefore may have been reluctant to participate due to inconvenience, were limited. Furthermore, the participant group was diverse regarding symptom burden and attitude towards DIHES.

As the findings are based on qualitative data, they may not be representative of all PwMS using DIHES. However, the study design provides an opportunity to explore issues in depth and to gain a nuanced understanding of the area of research. Care was taken to recruit diverse participants with regards to age, gender, type of MS, and educational level. The sample comprised few newly diagnosed participants (defined as being diagnosed less than 3 years prior) and no participants from ethnic minority groups. Studies on use of DIHES in the general population have found that ethnic minorities have lower rates of disclosure to HCPs regarding the types of DIHES they are using [45]. Hence, the findings of this study do not necessarily reflect where people from ethnic minorities seek information on DIHES. Further research examining the information needs of ethnic minorities is recommended. Another limitation of this study may stem from the fact that many of the participants have taken DIHES for a long time, and it may therefore be difficult for them to remember specific instances of information-seeking or dialogue with HCPs when they began using DIHES, which might have caused recall bias. To prevent the risk of recall bias, the interview guides were based on the participants’ personal answers from the initial survey.

## Conclusions

Use of DIHES among PwMS is widespread, and PwMS navigate between various sources of information regarding DIHES. In the present study, three main themes were identified regarding sources of information on DIHES among PwMS i) Engaging with HCPs regarding DIHES, ii) Social networks as a source of information regarding DIHES, and iii) Reliance on bodily sensations.

While some PwMS are comfortable navigating the available information, others may feel overwhelmed and confused. These findings suggest a need for better guidance of PwMS concerning DIHES and an openness among HCPs to engage in dialogue.

## Supplementary Information

**Additional file 1.** Interview guide. The semi-structured interview guide used in the study.

## Data Availability

The datasets used and/or analyzed during the current study are available from the corresponding author upon reasonable request.

## Abbreviations

MS: Multiple sclerosis
PwMS: People with multiple sclerosis
HCP: Healthcare professional
CAM: Complementary and alternative medicine
DIHES: Dietary and herbal supplements

## References

[1] Wallin MT, Culpepper WJ, Nichols E, Bhutta ZA, Gebrehiwot TT, Hay SI, et al. Global, regional, and national burden of multiple sclerosis 1990–2016: a systematic analysis for the global burden of disease study 2016. Lancet Neurol. 2019;18(3):269–85. 10.1016/S1474-4422(18)30443-5. 10.1016/S1474-4422(18)30443-5 PMC6372756 30679040

[2] Barin L, Salmen A, Disanto G, Babačić H, Calabrese P, Chan A, et al. The disease burden of multiple sclerosis from the individual and population perspective: which symptoms matter most? Mult Scler Relat Disord. 2018;25:112–21. 10.1016/j.msard.2018.07.013 30059895

[3] Chiaravalloti ND, DeLuca J. Cognitive impairment in multiple sclerosis. Lancet Neurol. 2008;7(12):1139–51. 10.1016/S1474-4422(08)70259-X. 10.1016/S1474-4422(08)70259-X 19007738

[4] Torkildsen Ø, Myhr K-M, Bø L (2016). Disease-modifying treatments for multiple sclerosis – a review of approved medications. Eur J Neurol.

[5] Kesselring J, Beer S (2005). Symptomatic therapy and neurorehabilitation in multiple sclerosis. Lancet Neurol.

[6] Loraschi A, Bellantonio P, Bortolon F, Capra R, Cavalla P, Costantino G, Lugaresi A, Martinelli V, Marrosu MG, Patti F, Rottoli M, Salvetti M, Sola P, Solaro C, Klersy C, Marino F, Zaffaroni M, Cosentino M (2016). Use of herbal remedies by multiple sclerosis patients: a nation-wide survey in Italy. Neurol Sci.

[7] Evans M, Paterson C, Wye L, Chapman R, Robinson J, Norton R, Bertschinger R (2011). Lifestyle and self-care advice within traditional acupuncture consultations: a qualitative observational study nested in a co-operative inquiry. J Altern Complement Med.

[8] Bergien SO, Petersen CM, Lynning M, Kristiansen M, Skovgaard L. Use of natural medicine and dietary supplements concomitant with conventional medicine among people with Multiple Sclerosis. Mult Scler RelatDisord. 2020;44:102197. 10.1016/j.msard.2020.102197. Epub 2020 May 23. 10.1016/j.msard.2020.102197 32531752

[9] National Center for Complementary and Integrative Health. Dietary and Herbal Supplements [Internet]. 2020. Available from: https://www.nccih.nih.gov/health/dietary-and-herbal-supplements. [cited 2021 Feb 17].

[10] The Danish Medical Agency. Natural medicinal products and vitamin and mineral products. 2017. Available from: https://laegemiddelstyrelsen.dk/en/special/natural-medicinal-products-and-vitamin-and-mineral-products/. [cited 2021 Feb 17].

[11] Zakaryan A, Martin IG (2012). Regulation of herbal dietary supplements: is there a better way?. Drug Inf J.

[12] Gotta M, Mayer CA, Huebner J (2018). Use of complementary and alternative medicine in patients with multiple sclerosis in Germany. Complement Ther Med.

[13] O’Connor K, Weinstock-Guttman B, Carl E, Kilanowski C, Zivadinov R, Ramanathan M (2012). Patterns of dietary and herbal supplement use by multiple sclerosis patients. J Neurol.

[14] Somerset M, Campbell R, Sharp DJ, Peters TJ (2001). What do people with MS want and expect from health-care services?. Health Expect.

[15] Pape K, Steffen F, Zipp F, Bittner S (2020). Supplementary medication in multiple sclerosis: real-world experience and potential interference with neurofilament light chain measurement. Mult Scler J Exp Transl Clin.

[16] Petersen MJ, Bergien SO, Staerk D. A systematic review of possible interactions for herbal medicines and dietary supplements used concomitantly with disease-modifying or symptom-alleviating multiple sclerosis drugs. Phytother Res. 2021. p. ptr.7050. 10.1002/ptr.7050 33624893

[17] Schwarz S, Knorr C, Geiger H, Flachenecker P (2008). Complementary and alternative medicine for multiple sclerosis. Mult Scler J.

[18] Hay MC, Strathmann C, Lieber E, Wick K, Giesser B (2008). Why patients go online: multiple sclerosis, the internet, and physician-patient communication. Neurologist.

[19] Marrie RA, Salter AR, Tyry T, Fox RJ, Cutter GR (2013). Preferred sources of health information in persons with multiple sclerosis: degree of trust and information sought. J Med Internet Res.

[20] Thorne S, Paterson B, Russell C, Schultz A (2002). Complementary/alternative medicine in chronic illness as informed self-care decision making. Int J Nurs Stud.

[21] Bishop M, Frain MP, Espinosa CT, Stenhoff DM (2009). Sources of information about multiple sclerosis: information seeking and personal, demographic, and MS variables. J Vocat Rehabil.

[22] Irvine A (2011). Duration, dominance and depth in telephone and face-to-face interviews: a comparative exploration. Int J Qual Methods.

[23] Lambert SD, Loiselle CG (2007). Health information—seeking behavior. Qual Health Res.

[24] Attride-Stirling J (2001). Thematic networks: an analytic tool for qualitative research. Qual Res.

[25] Arnett PA, Strober LB (2011). Cognitive and neurobehavioral features in multiple sclerosis. Expert Rev Neurother.

[26] Bol Y. Understanding fatigue in multiple sclerosis: from a psychological perspective [Internet]. Maastricht; Maastricht: NeuroPsych Publishers ; University Library, Universiteit Maastricht [host; 2010. Available from: http://pub.maastrichtuniversity.nl/ba1d75f0-6663-4688-b72c-80efd3d1a3d4. [cited 2021 Mar 12]

[27] Sundheds- og Ældreministeriet. Komitéloven [Internet]. Sect. 14 Sep 15, 2017. Available from: https://www.retsinformation.dk/eli/lta/2017/1083.

[28] Skovgaard L, Pedersen IK, Verhoef M. Use of bodily sensations as a risk assessment tool: exploring people with Multiple Sclerosis’ views on risks of negative interactions between herbal medicine and conventional drug therapies. BMC Complement Altern Med. 2014;14(1). Available from: https://bmccomplementalternmed.biomedcentral.com/articles/10.1186/1472-6882-14-59. [cited 2019 Mar 20] 10.1186/1472-6882-14-59 PMC3942187 24533750

[29] Leong EM, Semple SJ, Angley M, Siebert W, Petkov J, McKinnon RA (2009). Complementary and alternative medicines and dietary interventions in multiple sclerosis: what is being used in South Australia and why?. Complement Ther Med..

[30] Masullo L, Papas MA, Cotugna N, Baker S, Mahoney L, Trabulsi J (2015). Complementary and alternative medicine use and nutrient intake among individuals with multiple sclerosis in the United States. J Community Health.

[31] National Center for Complementary and Integrative Health (2018). Complementary, Alternative, or Integrative Health: What’s In a Name?.

[32] Synnot AJ, Hill SJ, Garner KA, Summers MP, Filippini G, Osborne RH, Shapland SDP, Colombo C, Mosconi P (2016). Online health information seeking: how people with multiple sclerosis find, assess and integrate treatment information to manage their health. Health Expect.

[33] Swar B, Hameed T, Reychav I (2017). Information overload, psychological ill-being, and behavioral intention to continue online healthcare information search. Comput Hum Behav.

[34] Jensen JD, King AJ, Carcioppolo N, Krakow M, Samadder NJ, Morgan S (2014). Comparing tailored and narrative worksite interventions at increasing colonoscopy adherence in adults 50–75: a randomized controlled trial. Soc Sci Med.

[35] Khaleel I, Wimmer BC, Peterson GM, Zaidi STR, Roehrer E, Cummings E, Lee K (2020). Health information overload among health consumers: a scoping review. Patient Educ Couns.

[36] Holst L, Wright D, Haavik S, Nordeng H (2009). The use and the user of herbal remedies during pregnancy. J Altern Complement Med.

[37] Nathan JP, Kudadjie-Gyamfi E, Halberstam L, Wright JT (2020). Consumers’ information-seeking behaviors on dietary supplements. Int Q Community Health Educ.

[38] Welz AN, Emberger-Klein A, Menrad K (2019). The importance of herbal medicine use in the German health-care system: prevalence, usage pattern, and influencing factors. BMC Health Serv Res.

[39] Welz AN, Emberger-Klein A, Menrad K (2018). Why people use herbal medicine: insights from a focus-group study in Germany. BMC Complement Altern Med.

[40] Weeks L, Balneaves LG, Paterson C, Verhoef M (2014). Decision-making about complementary and alternative medicine by cancer patients: integrative literature review. Open Med Peer Rev Indep Open Access J.

[41] Smith CA, Parton C, King M, Gallego G (2020). Parents’ experiences of information-seeking and decision-making regarding complementary medicine for children with autism spectrum disorder: a qualitative study. BMC Complement Med Ther.

[42] McClymont H, Gow J, Perry C (2014). The role of information search in seeking alternative treatment for back pain: a qualitative analysis. Chiropr Man Ther.

[43] Barnes LAJ, Barclay L, McCaffery K, Aslani P (2019). Complementary medicine products information-seeking by pregnant and breastfeeding women in Australia. Midwifery..

[44] Bhamra SK, Slater A, Howard C, Heinrich M, Johnson MRD (2019). Health care professionals’ personal and professional views of herbal medicines in the United Kingdom. Phytother Res.

[45] Gardiner P, Whelan J, White LF, Filippelli AC, Bharmal N, Kaptchuk TJ (2013). A systematic review of the prevalence of herb usage among racial/ethnic minorities in the United States. J Immigr Minor Health.

