# Supplementary material for: “I need personal experiences or some sort of documentation”: a qualitative study on where people with multiple sclerosis seek information on dietary and herbal supplements
Source: BMC Complement Med Ther. 2021 Aug 21;21:213. doi: 10.1186/s12906-021-03377-0 (PMC8379853; doi:10.1186/s12906-021-03377-0)

**Interview guide**

Sentences or words in *cursive* illustrate the participant’s answers from the cross-sectional survey or illustrate examples chosen specifically for one participant.

| Introduction |
| --- |
| **Thank you for taking the time to participate.**  **The interview will, among other things, be based on your answers from the questionnaire.**  **The interview is anonymous, and your name will not appear anywhere.**  **The interview will take approximately 40 min.**  **The interview is recorded, and I may take notes along the way.** |
| Reasons for using natural medicine and/or dietary supplements |
| **Would you like to start with saying a few words about what you think about using products such as vitamins, herbs and oils??**  **What types of natural medicine and dietary supplements do you currently use?**  In the survey, you answered that you took v*itamin D and calcium* – is that correct?  **Can you tell me about the time you started using *vitamin D and calcium*?**  Why did you start using *vitamin D and calcium*?  Is it still the reason you use it today?  Do you experience a tangible effect in your everyday life of *vitamin D and calcium* today?  **Have you ever considered using products like *magnesium and fish oil*?**  What would make you start using products like *magnesium and fish oil*?  **What would make you stop using *vitamin D and calcium*?** |
|  |
|  |
| Accessing information on natural medicine and/or dietary supplements *Note: The participant talks with healthcare professionals* |
| **You said that you used *vitamin d and calcium* because *of your MS*, how do you know that the product can be beneficial for this**  **When did you last hear and/or talk to anyone about natural medicine and dietary supplements**? **Can you tell me a little about what you read/talked about?**  Did you bring up the topic yourself/search for it?  Was the information easy to understand?  Have you been able to use what you heard/read for something afterwards?  Is this where you primarily get your knowledge about natural medicine and dietary supplements?  Do you ever talk to people in your network about natural medicine and dietary supplements?  Is there good and bad knowledge?  **Now I want to ask you more specifically about different sources of information. I will ask you to explain what makes it easy and/or difficult to access useful information about natural medicine and dietary supplements from these sources.**  **Do you think it is easy to get information about natural medicine and dietary supplements on *the internet*? Please explain with a few words why or why not?**  [If they do not think it is easy] Where do you think it could be easier to get information about natural medicine and dietary supplements, and why would this be easier?  How easy do you think it is to access useful information about natural medicine and dietary supplements from your *neurologist/general practitioner*? Please explain with a few words what makes it particularly easy or difficult?  **If a person who had recently been diagnosed with MS asked for your advice on natural medicine and dietary supplements and/or conventional medicine, what would you tell them?**  Where would you recommend this person to seek more information on the subject? |
|  |
|  |
|  |
|  |
|  |
|  |
|  |
|  |
|  |
|  |
|  |
|  |
|  |
| Communication with health care professionals about natural medicine and/or dietary supplements |
| **In the survey, you replied that you had talked to *your general practitioner and nurse at the sclerosis clinic* about the natural medicine and dietary supplements you took. Was it *vitamin D and calcium* you talked about?**  **Do you want to tell me about the last time you talked to a practitioner about this?**  What did you talk about?  Afterwards, was this knowledge useful for you?  Who brought up the topic?  How did you experience the situation?  **Have you talked to practitioners other than *your general practitioner and nurse at the sclerosis clinic* about natural medicine and dietary supplements?**  Do you want to tell me about that conversation?  Was there a difference in what you talked about compared to [the first mentioned]?  Was one of the experiences better than the other?  Who do you think it was easiest to talk to about natural medicine and dietary supplements?  [If the informant does not mention it] **Have you ever talked to alternative practitioners about natural medicine and dietary supplements?**  Why/why not?  **The responses from our survey show that few people talk to alternative practitioners about their use of natural medicine and dietary supplements, why do you think this is the case?**  **If you had to name the alternative practitioners you think would make the most sense to talk to about natural medicine and dietary supplements, who would it be?**  **If I mention practitioners like biopaths, naturopaths and psyhiopaths, do you know what they do?** |
|  |
|  |
| Knowledge about interaction between natural medicine and/or dietary supplements and conventional medicine |
|  |
| Presents he three pictures to the informant. **One person uses conventional medicine due to his/her MS (it can be both disease-modifying medicine and/or symptom-relieving medicine)** [picture A]**, the other person uses natural medicine and dietary supplements** [picture B]**, and the third person uses both natural medicine and/or dietary supplements and conventional medicine due to his/her MS** [picture C]**.**  **Which of the three people do you think is best treated for their MS and the associated symptoms, and who is the worst treated? Please explain your placement of the three people.**  Which practitioners do you think the three different people should talk to about their use of medicine?  **Do you think it is most likely that 1) the natural medicine and dietary supplements the person is taking are strengthening the body’s ability to absorb the conventional medicine that he/she is taking or 2) that it is impairing the body’s ability to absorb the conventional medicine that he/she is taking?** |
| Ending |
| **With this project, the Danish MS Society wants, among other things, to contribute to better support for citizens with MS in relation to their decision on/use of natural medicine and dietary supplements. If you were to give us three pieces of advice on how we best can help with this, what would you say?**  Is there any information you are missing on this subject?  What should we particularly focus on if we wanted people with MS to talk to the health care professionals about their use of natural medicine and dietary supplements?  **Is there something we did not cover in this interview that you would like to add or elaborate on?** |

Picture A


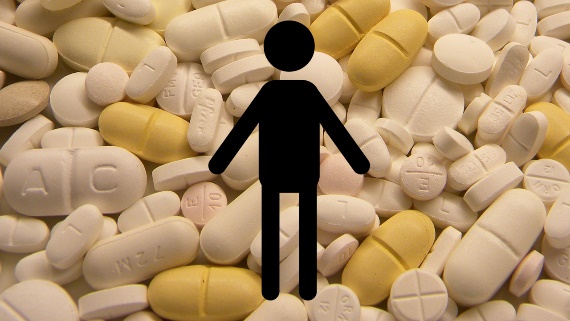


Picture B


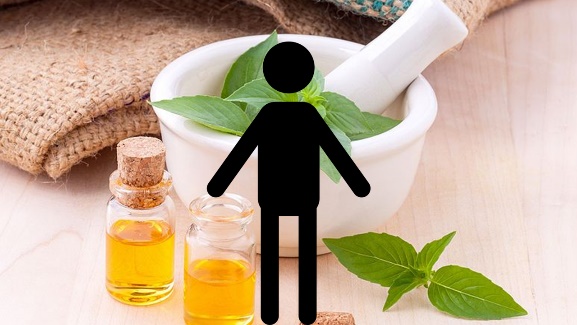


Picture C


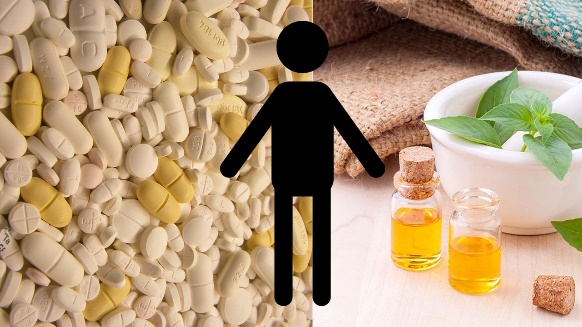

Supplement: Supplementary file 1 — Additional file 1. Interview guide. The semi-structured interview guide used in the study. [file 12906_2021_3377_MOESM1_ESM.docx]
